# Preparation of Novel Organic Polymer Semiconductor and Its Properties in Transistors through Collaborative Theoretical and Experimental Approaches

**DOI:** 10.3390/polym15224421

**Published:** 2023-11-16

**Authors:** Jinyang Chen, Yubing Ding, Jie Zhou, Na Li, Shiwei Ren, Minfeng Zeng

**Affiliations:** 1 Zhejiang Key Laboratory of Alternative Technologies for Fine Chemicals Process, Shaoxing University, Shaoxing 312000, China; 2023000069@usx.edu.cn (J.C.); ln991101@163.com (N.L.); 2 Zhuhai-Fudan Innovation Research Institute, Hengqin 519000, China; yubingding@fudan-zhuhai.org.cn

**Keywords:** conjugated polymer, semiconductor, optoelectronic devices, Stille coupling, high mobility, OTFT

## Abstract

Conjugated polymer semiconductors based on donor–acceptor structures are commonly employed as core materials for optoelectronic devices in the field of organic electronics. In this study, we designed and synthesized a novel acceptor unit thiophene-vinyl-diketopyrrolopyrrole, named TVDPP, based on a four-step organic synthesis procedure. Stille coupling reactions were applied with high yields of polymerization of TVDPP with fluorinated thiophene (FT) monomer. The molecular weight and thermal stability of the polymers were tested and showed high molecular weight and good thermal stability. Theoretical simulation calculations and 2D grazing-incidence wide-angle X-ray scattering (GIWAXS) tests verified the planarity of the material and excellent stacking properties, which are favorable for achieving high carrier mobility. Measurements based on the polymer as an organic thin film transistor (OTFT) device were carried out, and the mobility and on/off current ratio reached 0.383 cm^2^ V^−1^ s^−1^ and 10^4^, respectively, showing its great potential in organic optoelectronics.

## 1. Introduction

Organic semiconductor materials are potential materials for the preparation of optoelectronic devices due to their structure consisting of alternating single and double bonds, which allows for carrier transport across their conjugated systems [1]. Unlike inorganic semiconductors, organic semiconductor materials have good chemical modifiability, which is conducive to the preparation of novel materials with extensive structures to meet the application specifications and requirements of different devices [2,3]. At the same time, due to the preparation efficiency and flexibility of polymer materials, more and more research has been conducted on organic polymer systems [4]. After two decades of development, a variety of material systems have been developed and applied to a wide range of devices, including organic field-effect transistors, organic light-emitting devices, organic solar cells, sensors, memory storage, and organic thermoelectric and other functional devices [5,6,7,8,9,10]. In the case of organic transistors, for example, the primary performance indicators are electron and/or hole carrier transport and sensitive switching ratios [11,12]. The core material unit of organic transistors is organic semiconductor material, which is generally classified into donor–acceptor, donor–donor, and acceptor–acceptor based on the chemical composition [13,14]. Materials based on donor–acceptor are the most widely studied because they tend to be synthesized efficiently and with high yields [15,16,17]. At the same time, this type of architecture facilitates the flexible adjustment of the material’s frontline orbital energy levels, including the highest occupied molecular orbital (HOMO) energy level, the lowest unoccupied molecular orbital (LUMO) energy level, and the energy gap through the selection of appropriate donor and acceptor units and their ratio. Materials with appropriate energy levels bear a direct relationship to the type and speed of carrier migration [18]. 

**Figure 1:**
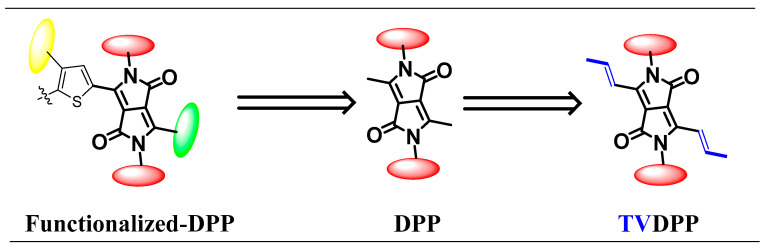
Schematic representation of the new structure TVDPP inspired by the classical DPP molecular structure.

Currently, the classical acceptor building blocks mostly contain imide structures, such as diketopyrrolopyrrole (DPP), isoindigo, perylene diimide, and so on [19]. New structures such as bithiophene imide, benzodifurandione-based oligo (p-phenylene vinylene), etc., have been developed, which are also capable of acting as acceptor units through strong electron-withdrawing groups and electron-attracting functional groups contained in the molecule [20,21,22]. It has to be mentioned that the design of completely new structures is both difficult and complicated, whereas chemical modification of classical structures is much more efficient and easier [2]. As shown in Figure 1 below, researchers have carried out a multifaceted and systematic modification strategy to improve the performance of the material, using classical DPP as the main candidate [23]. DPP monomers are inherently poorly soluble materials, which limits their material processing characteristics. Chemical modification of the N-position allows for easy introduction of side chains and also improves the solubility of the material. These include long aliphatic chains, alkyl chains with branches, siloxane chains, hydrocarbon chains with fluorine substituents, unsaturated hydrocarbon capped side chains, and so on [24,25,26,27]. The introduction of side chains increases the solubility of the material while at the same time facilitating the regulation of the stacking pattern and distance between the main chains. In some instances, the formation of intermolecular conformational locks also facilitates carrier transport. Modifications directed at the main chain are often associated with another component or multicomponent electron donor (acceptor) and not with direct modifications to the DPP backbone. This is because DPP itself has no unoccupied reactive sites and the properties of the material have to be modulated by modifications to the thiophene groups directly attached to it. The β-site of the thiophene can be conveniently used to insert strong electron-absorbing groups such as fluorine atoms, chlorine atoms, cyano groups, etc., as well as to introduce electron-donating groups such as methoxy and methyl groups. At the same time, the thiophene attached to the DPP substrate can be replaced by a variety of aromatic rings, including furan, bithiophene, benzene, and pyridine rings, so that a variety of structural materials can be prepared [28,29]. Our group has reported three types of DPP-based polymers this year and applied them as materials for hole transport, electron transport, and bipolar transport. The design concepts of these three structures are based on the introduction of copolymer units with electron-donating or electron-absorbing ability to modulate the device properties of the materials after the introduction of different side-chain groups to improve the solubility of the materials [30,31,32]. It is worth mentioning that up to now, there is still no effective method to directly synthesize DPP-based monomer structures without any aromatic ring [33]. Here, we propose a new synthetic idea and prepare a new structure of the molecular material and further introduce olefin to ensure the conjugated structure of the material.

## 2. Materials and Methods

Materials: Raw materials such as fumaroyl dichloride, 2-octyldodecylamine, triethylamine, etc., and organic solvents such as petroleum ether (PE), ethyl acetate (EA), dichloromethane (DCM), etc., were purchased from Sinopharm Chemical Reagent Company (Shanghai, China). (3,4-difluorothiophene-2,5-diyl)bis(trimethylstannane), abbreviated as 2FT-Sn, was acquired from SunaTech (Suzhou, China). Organic intermediates 2, 3, 4, TVDPP, and polymer PTVDPP-2FT were prepared as follows. All small molecules were characterized by Nuclear Magnetic Resonance (^1^H NMR, ^13^C NMR); their spectrograms are provided in the Supplementary Information (SI, Figures S1–S8).

Compound **2**: To a 250 mL two-necked flask was added 2-octyldodecylamine (7.44 g, 25 mmol) and triethylamine (2.02 g, 20 mmol) after sufficient nitrogen displacement. To the reaction system was added 100 mL of ultra-dry tetrahydrofuran solution (THF) under ice bath conditions, and the solution was stirred thoroughly for 30 min. Fumaryl chloride (1.53 g, 10 mmol) in THF was slowly added dropwise to the reaction flask, and the reaction mixture continued to be stirred for 2 h at room temperature before ending the reaction. The mixture was extracted by EA, and the organic phase was dried, filtered, concentrated, and then purified by column chromatography (V_EA_:V_PE_ = 1:3) to afford a white solid (compound **2**, 5.06 g, 75% yield). ^1^H NMR (400 MHz, CDCl_3_), δ H (ppm): 6.97 (s, 1H), 6.23 (s, 1H), 3.28 (t, J = 6.0 Hz, 2H), 1.55 (d, J = 7.2 Hz, 1H), 1.27 (d, J = 6.7 Hz, 51H), 0.88 (t, J = 6.6 Hz, 10H). ^13^C NMR (100 MHz, CDCl_3_), δ C (ppm): 164.45, 133.10, 43.32, 37.99, 31.92, 31.89, 31.87, 31.85, 30.02, 29.67, 29.64, 29.58, 29.35, 29.32, 26.67, 22.68, 22.67, 14.09. HR-MALDI-TOF m/z: [M+H]^+^ calculated for C44H87N2O2: 675.67676; found: 675.67671. 

Compound **3**: To a 150 mL round bottom flask was added intermediate 2 (4.45 g, 6.6 mmol) and isopropenyl acetate (50 mL) followed by p-toluenesulfonic acid (7 mL, 12% in acetic acid). The reaction system was refluxed for 5 h. The mixture was washed sequentially with 5% hydrochloric acid solution in water and saturated sodium bicarbonate solution. Purification of the collected product by column chromatography (V_EA_:V_PE_ = 1:5) afforded a brown liquid (compound **3**, 3.35 g, 67% yield). ^1^H NMR (400 MHz, CDCl_3_), δ H (ppm): 7.35 (s, 1H), 3.67 (d, J = 7.4 Hz, 2H), 2.42 (s, 3H), 1.66 (s, 1H), 1.25 (s, 38H), 0.88 (t, J = 6.7 Hz, 7H). ^13^C NMR (100 MHz, CDCl_3_), δ C (ppm): 173.57, 168.10, 134.35, 48.66, 38.10, 31.90, 31.87, 31.46, 29.98, 29.62, 29.57, 29.52, 29.33, 29.27, 26.43, 25.90, 22.66, 22.65, 14.06. HR-MALDI-TOF m/z: [M^+^H]^+^ calculated for C48H91N2O4: 759.69788; found: 759.69713. 

Compound **4**: Intermediate 3 (1.5 g, 2.0 mmol), triphenylphosphine (629.5 mg, 2.4 mmol), and pyridinium p-toluenesulfonate (PPTS, 251.3 mg, 1.0 mmol) were weighed into a thick-walled pressure-resistant flask, and a solvent mixture of THF and acetonitrile (3: 1, 40 mL total) was added. The reaction system was warmed to 100 °C overnight. DCM extraction followed by column chromatographic separation (V_EA_: V_PE_ = 1: 5) afforded a yellow oily liquid (compound **4**, 580.0 mg, 40% yield). ^1^H NMR (400 MHz, CDCl_3_), δ H (ppm): 3.39 (d, J = 7.5 Hz, 2H), 2.33 (s, 3H), 1.70 (d, J = 6.9 Hz, 1H), 1.25 (s, 46H), 0.88 (t, J = 6.8 Hz, 6H). ^13^C NMR (100 MHz, CDCl_3_), δ C (ppm): 162.57, 146.21, 108.42, 44.60, 38.14, 31.91, 31.88, 31.58, 29.98, 29.62, 29.60, 29.54, 29.34, 29.27, 26.56, 22.68, 22.65, 14.09, 12.98. HR-MALDI-TOF m/z: [M^+^H]^+^ calculated for C48H89N2O2: 725.69241; found: 725.69172. 

Compound TVDPP: Intermediate 4 (725.7 mg, 1.0 mmol), 5-bromothiophene-2-carboxaldehyde (477.6 mg, 2.5 mmol), L-proline (23.1 mg, 0.2 mmol), and triethylamine (4 mmol) were added to a 100 mL three-necked flask, followed by addition of a mixture of anhydrous toluene and methanol (5:1). The reaction mixture was protected by nitrogen at room temperature for 10 h. The reaction system was purified by column chromatography (V_DCM_: V_PE_ = 2:1) after extraction, and the resulting crude product was settled to afford a brown solid (TVDPP, 460.3 mg, 43% yield). ^1^H NMR (400 MHz, CDCl_3_), δ H (ppm): 8.87 (d, J = 15.4 Hz, 1H), 7.11–7.01 (m, 2H), 6.51 (d, J = 15.4 Hz, 1H), 3.64 (d, J = 7.2 Hz, 2H), 1.73 (q, J = 6.3 Hz, 1H), 1.38–1.20 (m, 35H), 0.86 (dt, J = 6.9, 4.2 Hz, 7H). ^13^C NMR (100 MHz, CDCl_3_), δ C (ppm): 161.56, 144.06, 143.88, 134.44, 131.58, 131.25, 115.69, 113.53, 109.67, 44.90, 38.83, 31.93, 31.91, 31.68, 30.01, 29.67, 29.59, 29.36, 29.31, 26.76, 22.69, 22.67, 14.10. HR-MALDI-TOF m/z: [M^+^H]^+^ calculated for C58H90Br2N2O2S2: 1071.48683; found: 1071.48743. 

Polymer PTVDPP-2FT: Under nitrogen protection, TVDPP (100.0 mg, 0.093 mmol), 2FT-Sn (41.5 mg, 0.093 mmol), 3,3,6,6-tetramethyl-9-(1,2,3,4-tetrahydroxybutyl)-4,5,7,9-t (Pd_2_dba_3_, 2.6 mg, 2.8 μmol), and tri-o-tolylphosphane (P(o-tol)_3_, 6.8 mg, 22.3 μmol) were sequentially added to a pre-dried 50 mL Schlenk flask. Anhydrous tetrahydrofuran solvent (7 mL) was added to the mixture, which was subsequently deoxygenated by a refrigerated pumping cycle under nitrogen. The reaction mixture was refluxed and allowed to react for 24 h at 120 °C with stirring. After returning to room temperature, the mixture was transferred to a beaker containing methanol (200 mL) and stirred for 2 h at room temperature. Further filtration afforded solid particles, which were subsequently loaded into a Soxhlet extractor. Sequentially, methanol, acetone, ethyl acetate, and hexane were utilized over 10 h to remove the oligomers. The extract was subsequently run with chloroform and concentrated to remove the solvent. Precipitation was again carried out with anhydrous methanol (150 mL) for 3 h. The solid was filtered and subsequently dried under vacuum (90 °C) to give a black polymer (PTVDPP-2FT, 89% yield).

Organic thin film transistor (OTFT): devices were fabricated on 0.6 cm × 0.6 cm SiO_2_/Si substrates with a bottom-gate-bottom-contact (BGBC) configuration. The SiO_2_/Si substrates were blown dry with nitrogen after ultrasonic cleaning in deionized water, ethanol, and isopropanol for 7 min each, followed by deposition of the source and drain electrodes by thermal evaporation. The channel width/length of the field effect transistor devices were 1400/30 µm, respectively. PTVDPP-2FT was pre-dissolved in chlorobenzene at high temperature (60 °C). The polymer films were deposited onto the substrates by spin-coating at a fixed spin-coating rate of 3000 rpm under ambient conditions. Subsequently, the polymer films were annealed at 150 °C for 10 min to remove the corresponding solvents.

Characterization: The molecular weight and degree of dispersion of the PTVDPP-2FT was evaluated by high temperature gel permeation chromatography (PL-GPC220, Agilent, Santa Clara, CA, USA). Trichlorobenzene is used for the eluent, and polystyrene is used as a standard. A solution at a concentration of 0.1 mg/mL was passed through the column (2× PLgel 10 um MIXED-B) at a flow rate of 1.0 mL/min and tested at 150 °C. The thermal stability of the solid powders was measured by a thermal gravimetric analyzer (METTLER TGA, Greifensee, Switzerland) in a nitrogen atmosphere with a heating rate of 10 °C per minute at temperatures from 50 to 550 °C. Differential scanning calorimetry (NETZSCH, Waldkraiburg, Germany) was used to measure the reaction heat of the solid powders in a nitrogen atmosphere with a heating rate of 10 °C per minute, including the first heating process 1 (from room temperature to 250 °C), the first cooling process 2 (from 250 °C to room temperature) and the second heating process 3 (from room temperature to 250 °C). Elemental analysis is measured by the CHN mode of the organic elemental analyzer (FlashSmart, Thermo Scientific, Waltham, MA, USA). Photochemical characterization was performed on a UV-visible spectrometer (Cary5000, Agilent, Santa Clara, CA, USA). The concentration of the solution is approximately 0.3 mg/mL in chloroform. The polymer semiconductor solution was spin-coated onto a pre-cleaned quartz plate and then thermally annealed at 150 °C for 20 min. Electrochemical tests were carried out in acetonitrile solutions containing tetrabutylammonium hexafluorophosphate as an electrolyte. A Ag/AgCl electrode (Ag in a 0.01 mol/L KCl), glassy carbon electrode, and platinum electrode were applied as the reference electrode, working electrode, and counter electrode, respectively. A drop of 5 µL of polymer chloroform solution was deposited with a pipette gun onto a glassy carbon electrode and allowed to evaporate slowly (the circular hole of the electrode was 4 mm in diameter). OTFT transfer and output characterizations were carried out in a nitrogen glove box using a Keithley 4200 semiconductor characterization system (Keithley, Cleveland, OH, USA). 2D-GIWAXS results were obtained on the 14B/15U beamline station of the Shanghai Synchrotron Radiation Facility. Atomic force microscopy measurements were carried out using a Nanoscope V instrument (Bruker, Mannheim, Germany). These samples were identical to those used in OTFT performance analysis.

## 3. Results

### 3.1. Synthesis Routes to Polymer PTVDPP-2FT through Stille Coupling Polymerization

**Scheme 1:**
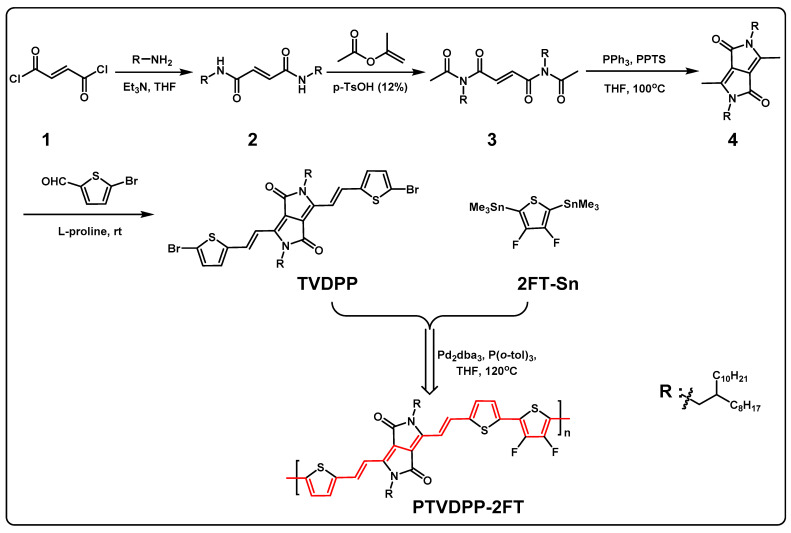
Preparation of the monomer and the synthetic process based on the palladium-catalyzed coupling polymerization reaction route, where alternating single and double bonds are shown in red.

Scheme 1 enumerates the synthetic routes for the preparation of polymers from monomers; the steps and details of the synthesis of the raw materials and intermediates numbered 1 to 4 are described above. The intermediate 4 with two five-membered heterocycles obtained by cyclization show excellent planarity and a fused structure. In addition to this, the intra-molecular inclusion of two carbonyl groups (C=O) leads to strong electron-withdrawing properties of this type of material, which is beneficial for the formation of polymers with alternating acceptor–donor type arrangements. The nitrogen positions of the side chains are introduced with long alkyl chains, which on the one hand increases the solubility and subsequent processability of the material, and on the other hand does not drastically affect the intermolecular stacking distances. TVDPP, further prepared via step 4, is a promising precursor for the Stille coupling reaction. The conformation of the olefin in TVDPP is adopted in the trans-form, which was obtained by the calculation of the coupling constants in the NMR pattern. The insertion of the olefin itself has no negative effect on the planarity of the material, while the rigid structure facilitates the efficient transport of carriers. It is worth mentioning that the introduction of olefins has a significant effect on the increase of the conjugation length of the monomer and the polymer. The monomer TVDPP can be easily copolymerized with the bis-trimethyltin-containing monomer 2FT under palladium catalysis. The schematic representation of the polymer with alternating ordering of the single and double bonds is highlighted in red. 

**Figure 2:**
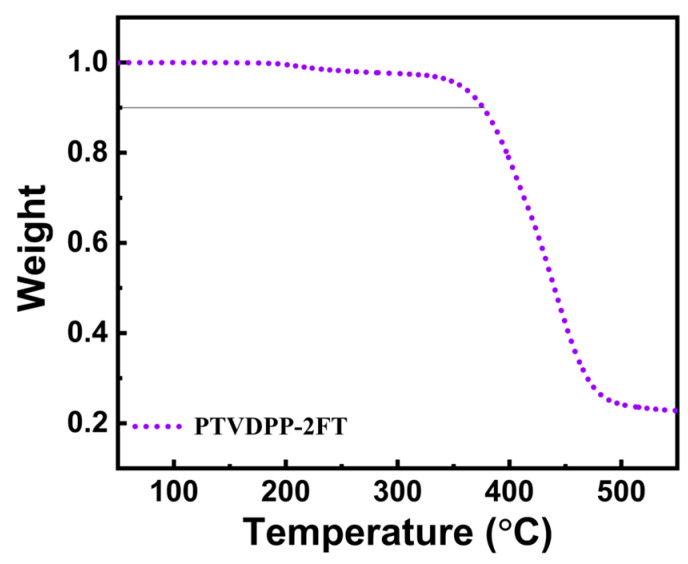
Thermal gravity analysis of polymer PTVDPP-2FT.

Common polymerization reactions rely on chlorobenzene as the reaction solvent, which is related to the poor solubility of the material. In this study, we utilized tetrahydrofuran (THF) as the solvent for the polymerization reaction and successfully prepared materials of suitable molecular weight. THF is a green and environmentally clean solvent; environmental and human hazards are thus reduced as compared to toxic reagents containing chlorine. The second method of molecular weight control is based on the regulation of the polymerization time. The molecular weight of the macromolecules increases as the polymerization time increases, causing them to precipitate in the THF solvent, which slows down and stops the reaction. The molecular weights of the polymers were evaluated, and the number average molecular weight (Mn) and weight average molecular weight (Mw) were found to be 16.97 kDa and 35.78 kDa. The average degree of polymerization of the final material was found to be 16 and 33 based on the ratio of the Mn or Mw value of the polymer to the molecular weight of the smallest repeating unit, respectively. The dispersion (Đ) of the material was found to be good, with a value of only 2.10 based on the ratio of Mw to Mn. It can be found that most of the polymers used for organic semiconductor applications show dispersions in the range of 2.0−3.5, which is related to their degree of polymerization and the purification methods. Polymers with extremely long and average length chains are more chaotic, while average-length polymer chains cannot be decontaminated by the Soxhlet extraction technique due to their poor solubility in normal organic solvents. The specific results of the molecular weight of the polymers are presented in Figure S9. The purity of the final product can be deduced from the elemental analysis of the polymer. The proportions of the three elements C, H, and N contained in the polymer are within tolerance of the theoretical composition of the proportions of the elements that should be expected in the smallest repeating unit (Table 1). The thermal behavior of the polymer remains almost unchanged at 350 °C, with a loss of less than 0.05% of the initial mass fractions, as shown in Figure 2. The temperature at which it loses 10% of its mass is 375 °C, demonstrating its good thermal stability. The results of differential scanning calorimetry (DSC) showed no significant phase change in the range of 30−250 °C (Figure S10).

### 3.2. Density Functional Theory Calculation

Polymers are difficult to culture into crystals; we used density functional theory (DFT) to study the theoretical configurations of the materials. The theoretical calculations regarding the planarity of the materials with respect to the front molecular orbital energy levels are performed with geometry optimization under the B3LYP/6-31G(d) basis group [34,35]. In order to simplify the calculations and reduce the computational cost, the long alkyl chain at the N-position was simplified to a short methyl chain. The non-conjugated composition of the side chains barely affects the energy levels of the structures conjugated on the main chain. On the other hand, the investigation used dimers as samples to simulate the long conjugated structures, a method accepted in many studies, which facilitates a significant reduction of the computational time. 

**Figure 3:**
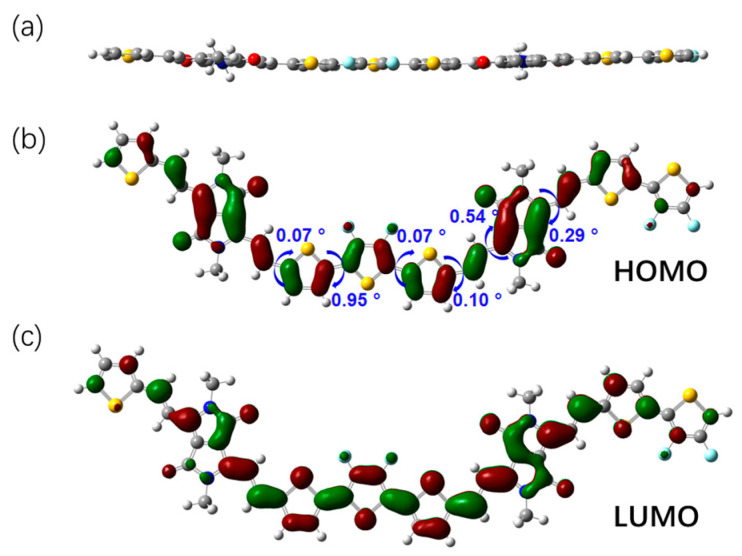
Optimized conjugated backbone conformation of the methyl-substituted dimer of PTVDPP-2FT: (**a**) top view; (**b**) side view and frontier molecular orbital HOMO map; (**c**) LUMO map.

As shown in the top view of the material in Figure 3a, the dimer structure exhibits good planarity, which is consistent with the envisaged characterization of the rigidity of the fused five-membered ring and the double bond. The thiophene was used as a bridging group to the two olefinic structural units, and the combined trithiophene-like structures show a head-to-tail linkage pattern. We measured the dihedral angles between thiophene and thiophene, which were overall less than 1°; this fully ensures the planar regularity of the polymer system. The geometrical configuration of the DFT-optimized dimer is almost planar. The good co-planarity effectively enhances π−π stacking and improves intramolecular and intermolecular charge transport and thus facilitates the achievement of high mobility. Both HOMO and LUMO are fully delocalized on the dual DPP acceptor moiety and the π−conjugated bridge donor segment. HOMO mainly occupies the double bonds of the molecule and is well separated from the domains throughout the horizontal backbone of the molecule. LUMO is mainly located in the DPP unit of the molecular chain and shows a strong dispersion; therefore, it is more beneficial for the transport of hole carriers. According to theoretical calculations, the HOMO and LUMO energy levels of the dimer are −4.73 eV and −3.10 eV, respectively, corresponding to an energy gap of 1.63 eV.

### 3.3. Photochemical Properties and Electrochemical Properties

**Figure 4:**
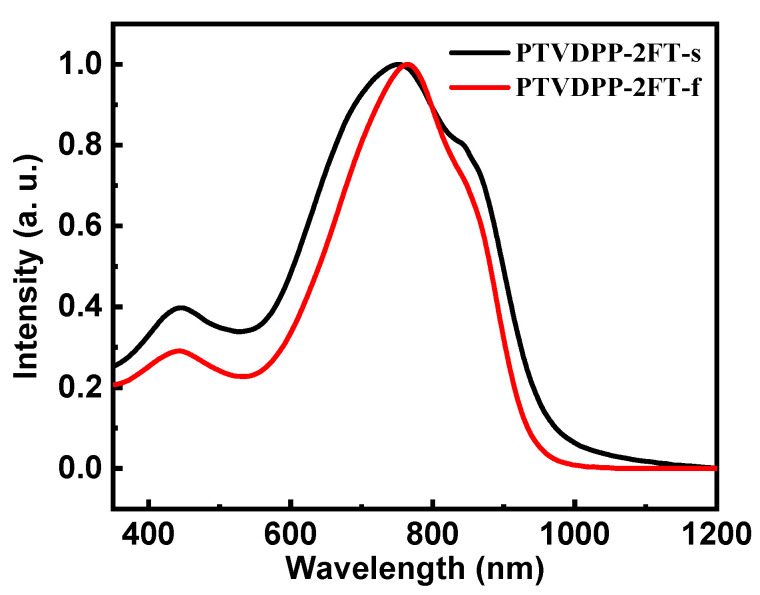
UV-vis absorption patterns of PTVDPP-2FT. Black line: solution state; red line: film state.

In order to investigate the photochemical absorption properties of the polymer, its UV-visible absorption profiles in chloroform solution and in the solid phase of the film were tested. PTVDPP-2FT, similar to most polymers with structures based on donor–acceptor, shows a dual absorption band. The lower wavelength absorption band is between 350 nm and 450 nm, which is due to the π−π* transition (Figure 4). The higher wavelength low-energy absorption is between 600 nm and 1000 nm, which is the result of charge transfer between the donor and acceptor within the molecule. The two maximum absorption peaks presented in the solution state occur at 445 nm and 750 nm, respectively, and show almost no absorption beyond 1000 nm. Compared to the absorption in the solution phase, the maximum absorption peak of the polymer in the solid film state undergoes a redshift to 765 nm, which is related to the stacking of the polymer in the solid state. Based on the position of the onset absorption peak, the bandgap of the polymer can be estimated to be 1.35 eV. The narrow bandgap is determined by the fact that it features long conjugated main chains, which is favorable for the formation of materials with high carrier mobility.

**Figure 5:**
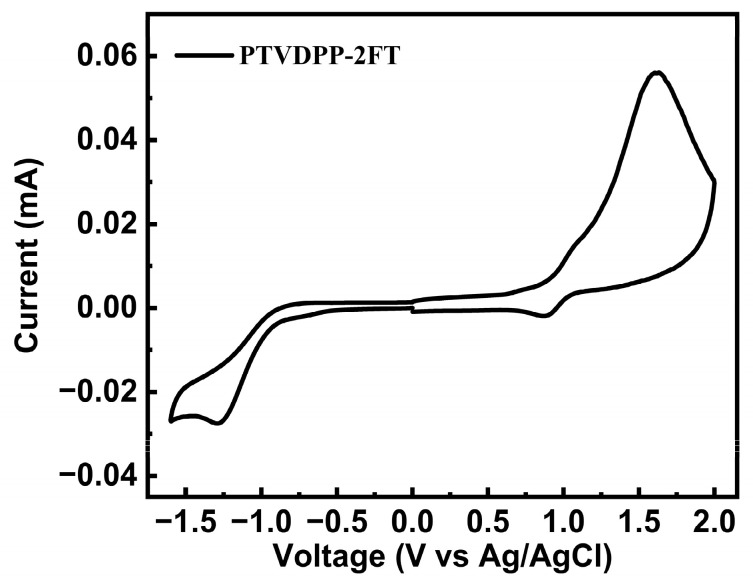
Cyclic voltammetry patterns of polymer PTVDPP-2FT film.

In order to further measure the redox properties of the materials, the polymers were measured in the thin film state using cyclic voltammetry (Figure 5). The material shows a more pronounced oxidation peak compared to its reduction curve, which to some extent indicates that the material has a more prominent ability to be oxidized and is suitable for use as the P-type hole transport material. The onset of the oxidation peak is at 1.03 V, which corresponds to the HOMO energy level of the material being near −5.47 eV. Compared to the oxidation curve, its reduction peak exhibits quasi-reversibility. Based on the onset of the reduction peak, the LUMO energy level can be inferred to be −3.52 eV.

### 3.4. OTFT Device Performance

**Figure 6:**
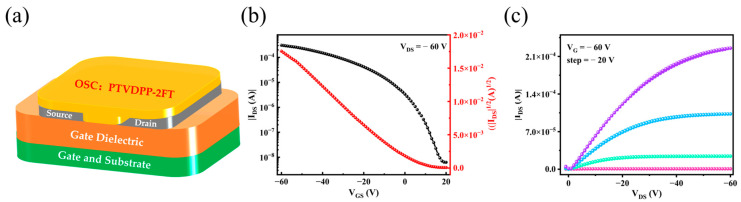
(**a**) Device configuration of BGBC OTFTs; (**b**,**c**) transfer and output curve of PTVDPP-2FT-based device. The pink, green, blue, and purple lines in (**c**) represent voltages of 0 V, −20 V, −40 V, and −60 V, respectively.

In order to characterize the charge transport behavior of these materials, we fabricated bottom-gate-bottom-contact (BGBC)-structured OTFT devices based on PTVDPP-2FT, and the corresponding device configuration is shown in Figure 6a. The deliberate selection of gold (Au) electrodes as part of the device is related to the fact that the HOMO energy levels of the polymer, obtained through electrochemical measurements, match those of the Au electrodes. Owing to the poor solubility of the polymer in THF solvent and the volatility of THF itself, the prepared polymer films were not homogeneous. On the other hand, the solubility of the polymer in chlorobenzene was much better, approximately 10 mg/mL, and therefore chlorobenzene was finally adopted. The saturation mobility (*µ*) is calculated as follows:

$$µ={\left(\frac{\partial \surd {|I}_{DS}|}{\partial V_{GS}}\right)}^{2}\;\cdot \;\frac{2L}{WCi}$$

where *I_DS_* and *V_GS_* are the source-drain current and gate voltage, respectively; W and L are the channel width and length, respectively; and Ci is the capacitance per unit area of the gate dielectric layer.

Table 2 summarizes the hole mobility extracted from the transfer characteristic curve (Figure 6b). The average hole mobility based on ten sets of devices is in the vicinity of 0.356 cm^2^ V^−1^ s^−1^, which is excellent for P-type materials. The maximum hole mobility of the polymer is 0.383 cm^2^ V^−1^ s^−1^, which is achieved by modulation, including film thickness and annealing temperature. In addition to excellent mobility, the device shows low threshold voltage (V_th_) and high switching ratio (I_on_/I_off_), indicating excellent device performance [36]. The output behaviors of the PTVDPP-2FT-based devices are shown in Figure 6c.

### 3.5. Morphological Analysis of PTVDPP-2FT Films

**Figure 7:**
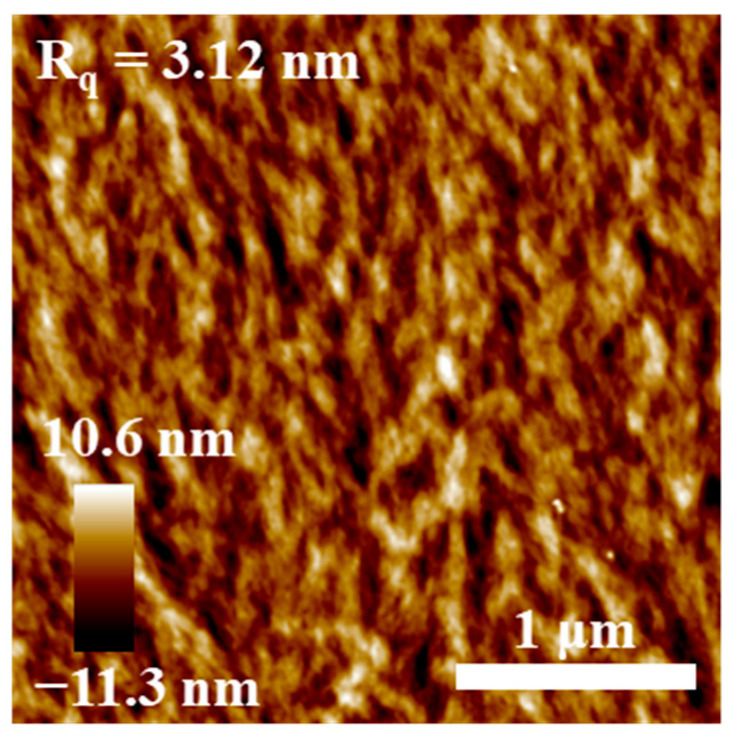
AFM height image of annealed film of polymer PTVDPP-2FT.

We investigated the relationship between polymer film morphology and device performance using atomic force microscopy (AFM) images. Figure 7 and Figure S11 show the AFM height and phase images of the annealed polymer film at optimal OTFT device performance conditions. We calculated the root-mean-square (RMS) roughness of the annealed film, and the surface roughness of PTVDPP-2FT was 3.12 nm. Combined with the AFM phase image, the polymer annealed films show good continuity without significant phase separation, which facilitates the achievement of high carrier mobility.

### 3.6. Grazing Incidence X-ray Diffraction of the Polymer Films

**Figure 8:**
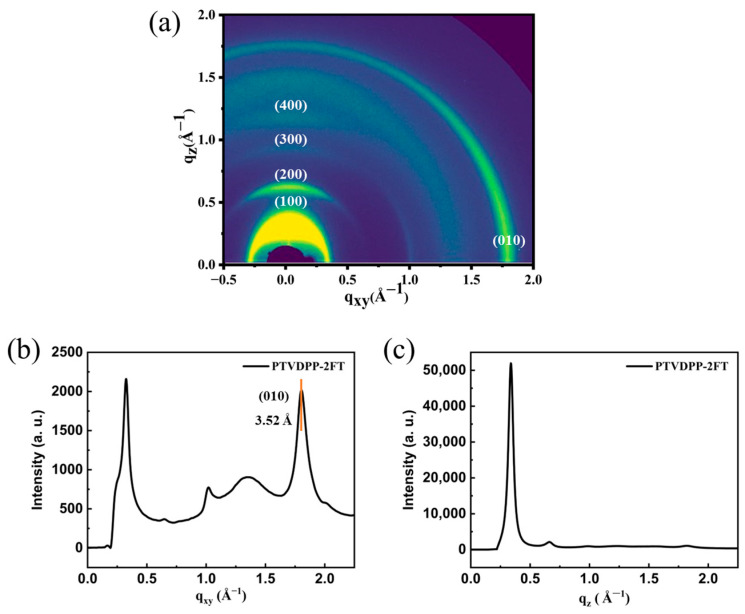
2D-GIWAXS pattern (**a**) and 1D-GIWAXS profile (**b**,**c**) of PTVDPP-2FT annealed film.

We investigated the relationship between the crystallinity of polymer film and the performance of the OTFT device using 2D-GIWAXS tests. As can be seen in Figure 8a, the polymer shows fourth-order diffraction peaks, including (100), (200), (300), and (400), and the polymer film adopts an edge-on stacking mode. Substituting the angle corresponding to the (100) diffraction peak of the polymer PTVDPP-2FT into the Bragg’s equation dsinθ = nλ, the d–d distance is calculated to be 27.14 Å. Based on the out-of-plane (010) diffraction peaks, the π−π stacking distance of the PTVDPP-2FT is estimated to be 3.52 Å (Figure 8b,c). The structure also uses FT as the monomer, and during its polymerization with DPP, we measured a stacking distance of approximately 3.95 Å [32]. Most polymer systems based on the classical DPP structure tend to exhibit greater stacking distances, in the range of 3.7−4.0 Å [37]. The tight π−π stacking is beneficial for carrier transport and achieves high mobility of the polymer film [11].

## 4. Discussion

To design better materials with excellent device properties, expanding the variety of molecular libraries is often a straightforward and effective approach [12,38]. A typical issue facing polymeric building block materials with donor–acceptor structures is the severe lack of available acceptor species [20]. Some representative structures have been developed and show good carrier mobility [39,40,41]. In order to further enrich the diversity of materials, the aim of this study was to break the classical single bond connecting DPP to thiophene and to introduce additional olefin. The route for the synthesis of the monomers is not complicated, and the chemical reagents used and the reaction conditions are not demanding. Polymer PTVDPP-2FT can be prepared by a coupling polymerization reaction with fluorinated thiophene and shows good properties in P-type materials. Next, more typical electron donor units can be applied to our materials, and polymers with a variety of structures can be formed. Not limited to P-type unipolar transport, the design and preparation of ambipolar materials as well as N-type materials with high electron mobility are also future fields of application for the system. We hope that the new molecules can be used as novel potential units for expanding the field of OTFT.

## 5. Conclusions

In this work, the synthetic route of monomer TVDPP based on a four-step preparation is explored and reported. Distinguishing from the classical structure DPP, the monomer features a longer conjugated structure and a more planar structure. Further, Sn-containing electron donor units can be conveniently introduced by Stille coupling polymerization to prepare macromolecules. The absence of chlorine-containing reagents in the entire polymer preparation process is favorable for the development of green polymerization methods. Polymer PTVDPP-2FT exhibits excellent thermodynamic stability and solubility. Results based on theoretical calculations show that it exhibits a particularly planar structure, which facilitates the transfer of holes through its main chain. The results based on GIWAXS revealed a tight stacking pattern between chains, which on the other hand facilitates the hopping transfer of carriers. Ultimately, OTFT devices based on this material show hole transfer rates up to 0.383 cm^2^ V^−1^ s^−1^ and typical P-type transport characteristics. More materials employing this monomer as a core structural unit are being prepared and tested in our lab for use in optoelectronic devices.

## Figures and Tables

**Table 1:** Molecular weight of the polymer and the ratio of the three elements it contains.

	Mn	Mw	Đ	C	H	N
				(%)	(%)	(%)
PTVDPP-2FT	16,970	35,780	2.10	72.84	8.28	2.58
repeating unit ^1^	N/A	N/A	N/A	72.33	8.81	2.72

^1^ The molecular chemical formula is C_62_H_90_F_2_N_2_O_2_S_3_.

**Table 2:** Hole transport properties of PTVDPP-2FT-based OTFT devices.

Material	Coating Speed (mm/s)	Annealing Temperature (°C)	Max Hole Mobilities (cm^2^/(V s))	Hole Mobilities ^1^ (cm^2^/(V s))	V_th_ (V)	I_on_/I_off_
PTVDPP-2FT	3000	150	0.383	0.356	4.10	4.89 × 10^4^

^1^ Measurement results based on ten OTFT devices.

## Data Availability

Data are contained within the article and Supplementary Materials.

## Supplementary Materials

The following supporting information can be downloaded at: https://www.mdpi.com/article/10.3390/polym15224421/s1, Figure S1: ^1^H NMR spectra of compound **2**; Figure S2: ^13^C NMR spectra of compound **2**; Figure S3: ^1^H NMR spectra of compound **3**; Figure S4: ^13^C NMR spectra of compound **3**; Figure S5: ^1^H NMR spectra of compound **4**; Figure S6: ^13^C NMR spectra of compound **4**; Figure S7: ^1^H NMR spectra of compound TVDPP; Figure S8: ^13^C NMR spectra of compound TVDPP; Figure S9: GPC data for the polymer; Figure S10: Differential scanning calorimetry of polymer PTVDPP-2FT. Figure S11: AFM phase image of annealed film of polymer PTVDPP-2FT.
