# Supplementary material for: Preparation of Novel Organic Polymer Semiconductor and Its Properties in Transistors through Collaborative Theoretical and Experimental Approaches
Source: Polymers (Basel). 2023 Nov 16;15(22):4421. doi: 10.3390/polym15224421 (PMC10674425; doi:10.3390/polym15224421)

# Supplementary Information

**Figure S1**. $^{1}$H NMR spectra of compound 2

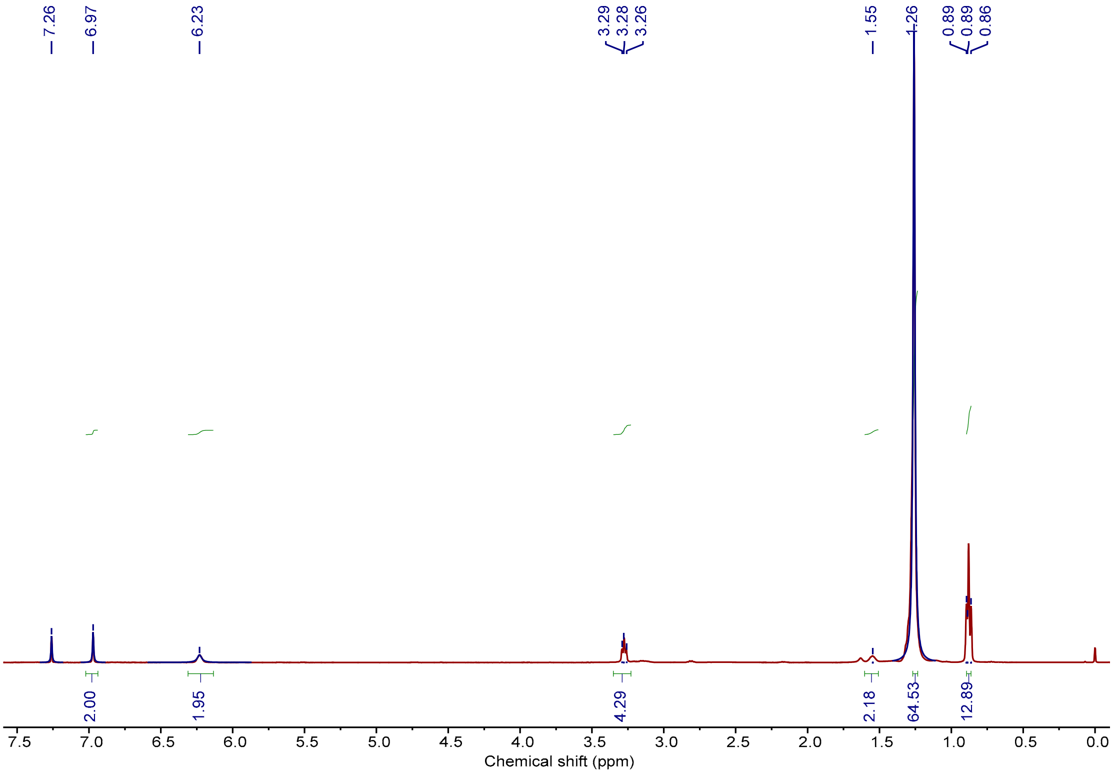


**Figure S2**. [1]C NMR spectra of compound 2

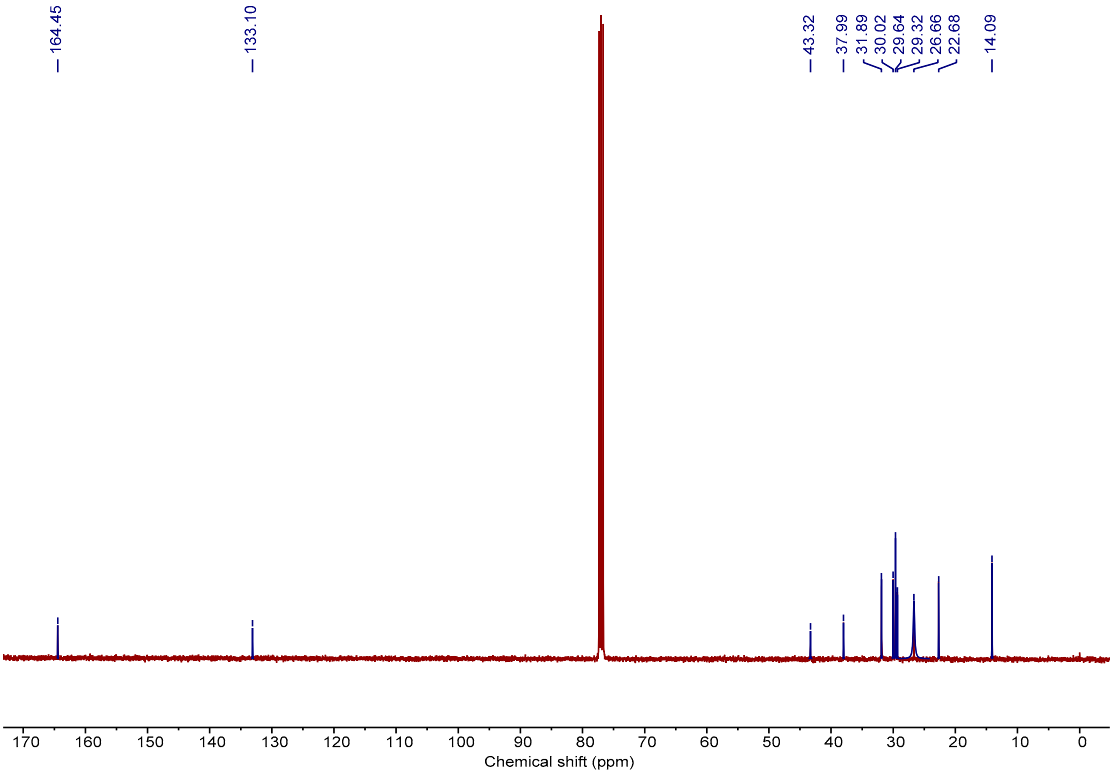

**Figure S3**. $^{1}$H NMR spectra of compound 3

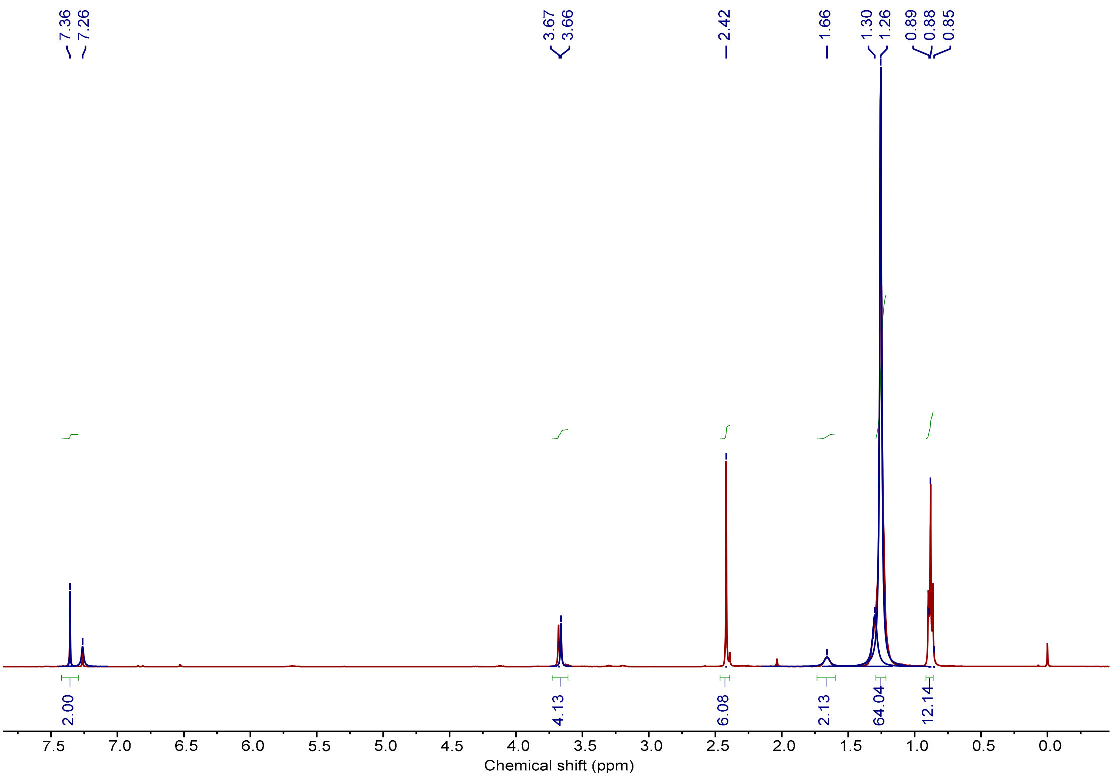


**Figure S4**. $^{1}$C NMR spectra of compound 3

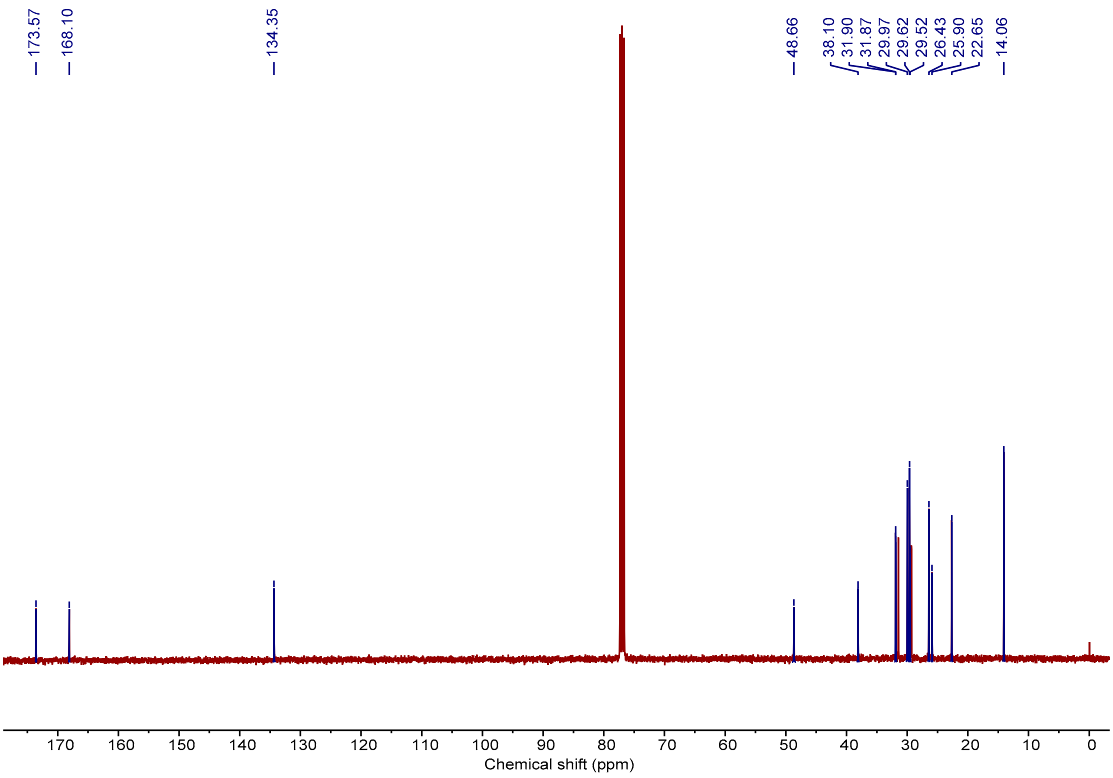

**Figure S5**. ¹H NMR spectra of compound 4

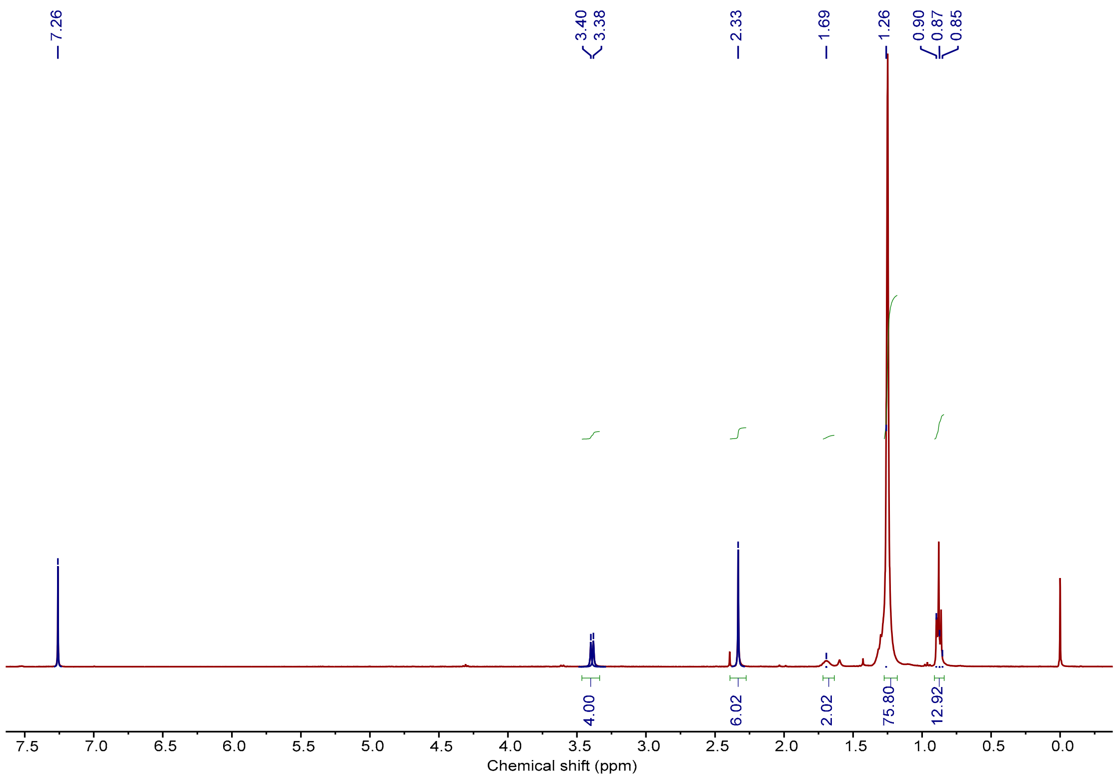


**Figure S6**. ¹C NMR spectra of compound 4

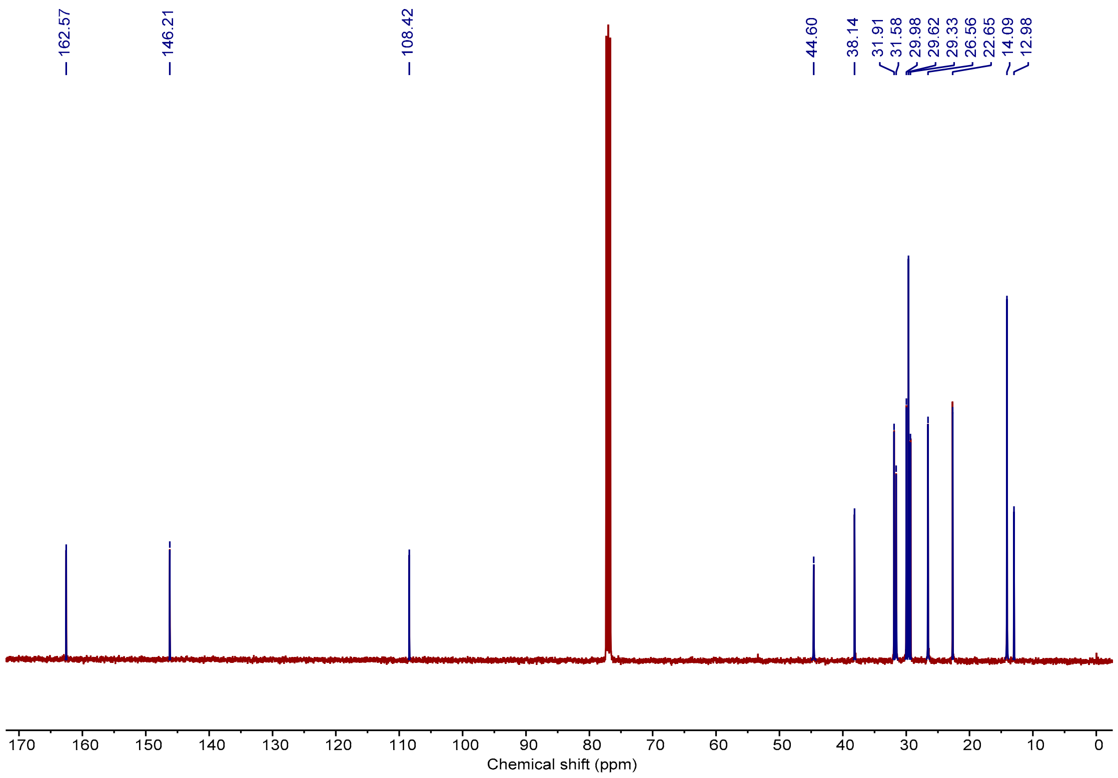

**Figure S7**. $^{1}$H NMR spectra of compound TVDPP

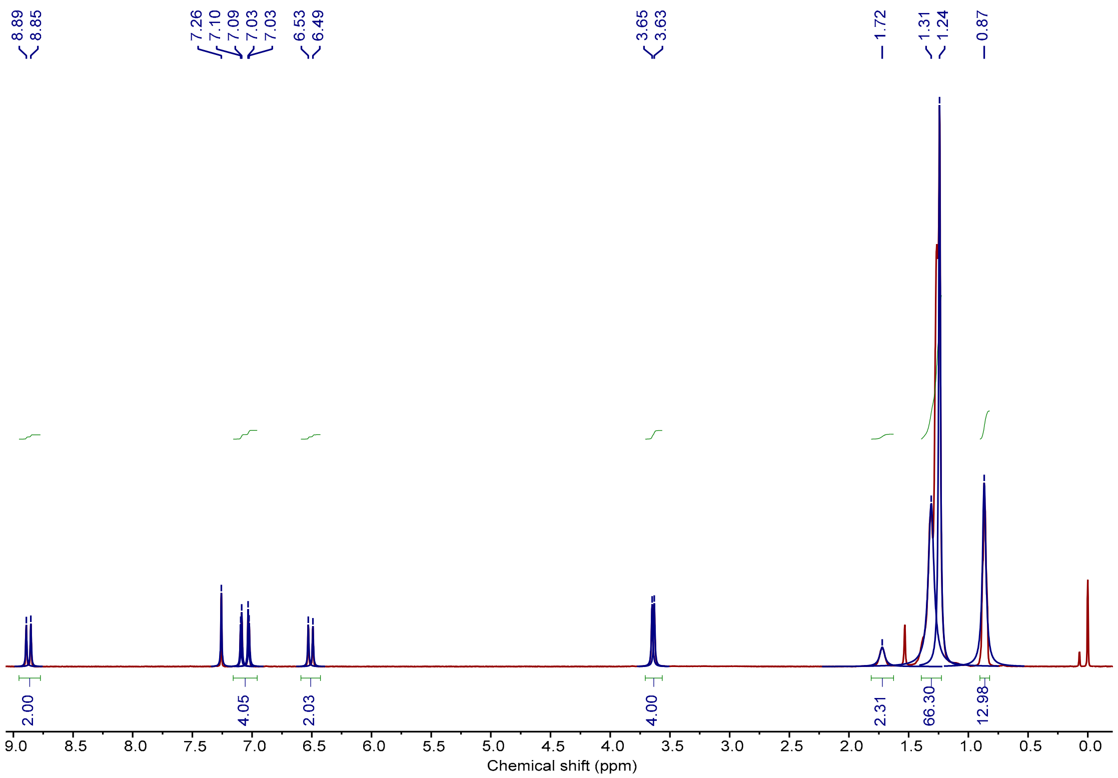


**Figure S8**. $^{13}$C NMR spectra of compound TVDPP

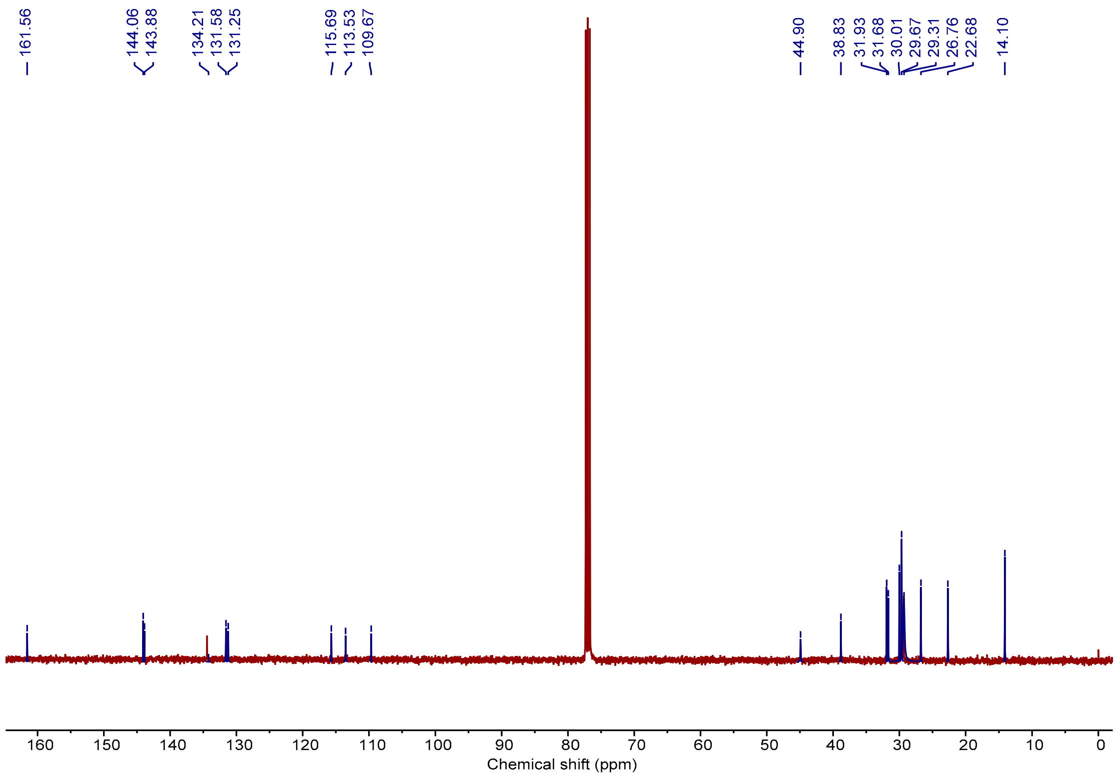

**Figure S9.** GPC data (cumulative percent curves and molecular weight distribution) for the polymer.

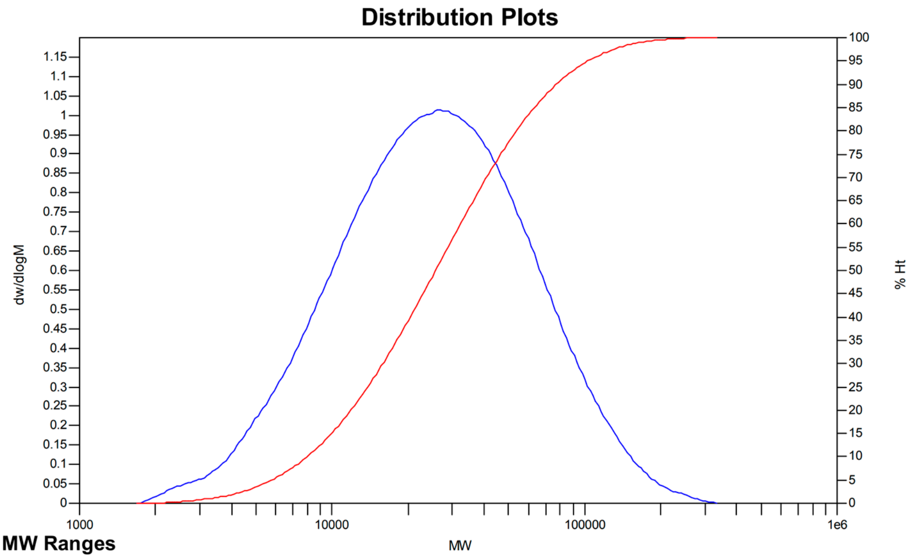


**Figure S10:** Differential scanning calorimetry of polymer PTVDPP-2FT.

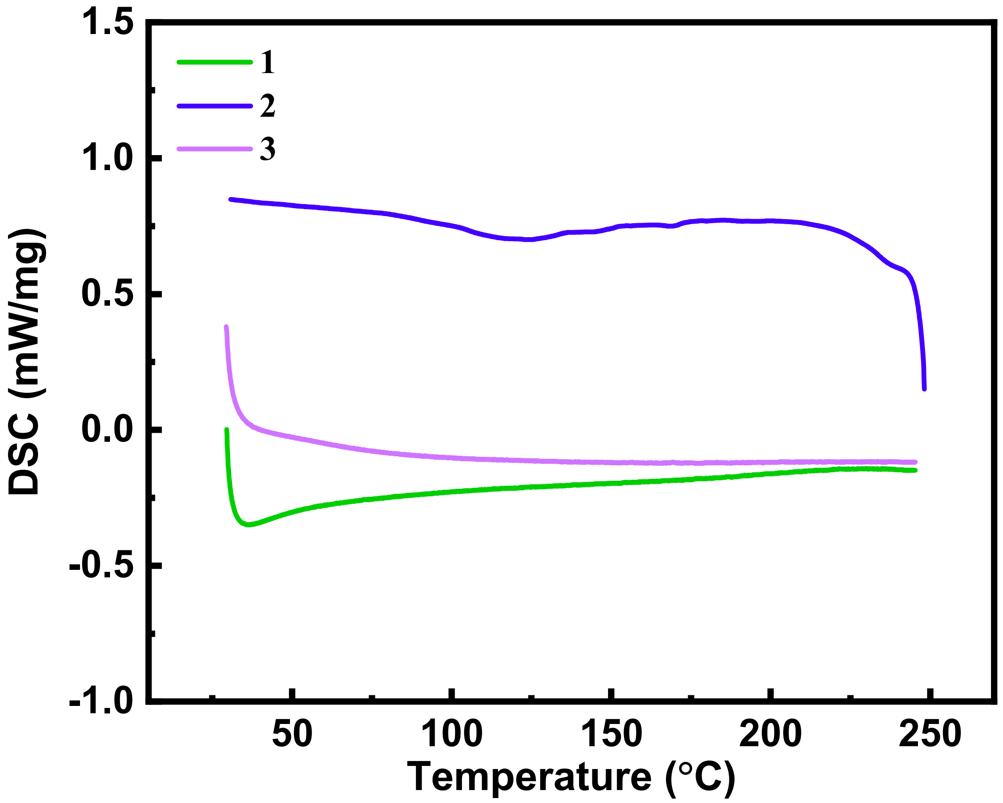

**Figure S11:** AFM phase image of annealed film of polymer PTVDPP-2FT.

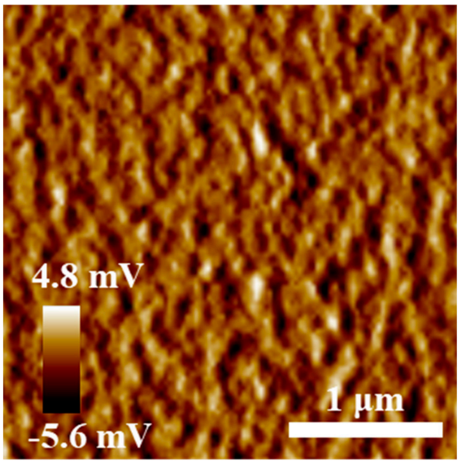

Supplement: Supplementary file 1 [file polymers-15-04421-s001.zip › polymers-2719950-supplementary.pdf]
